# Lipid metabolism and inflammation as key drivers in preterm birth: A comprehensive analysis

**DOI:** 10.1002/ijgo.70285

**Published:** 2025-06-07

**Authors:** Shu Xiao, ChaoChao Wei

**Affiliations:** ^1^ Obstetrics and Gynecology, Xiangya Hospital Central South University Changsha Hunan China; ^2^ Department of Oncology, Xiangya Hospital Central South University Changsha China; ^3^ Department of Pulmonary and Critical Care Medicine Affiliated Hainan Hospital of Hainan Medical University Haikou China; ^4^ NHC Key Laboratory of Tropical Disease Control Hainan Medical University Haikou China

**Keywords:** circulating inflammatory factors, Lipidome, Mendelian randomization, preterm birth

## Abstract

**Objective:**

To investigate the alterations in lipid metabolites and circulating inflammatory factors associated with preterm birth, elucidate their interactions, and uncover the underlying pathophysiologic mechanisms. By identifying potential predictive biomarkers and informing the development of effective therapeutic interventions, this research seeks to improve maternal and neonatal health outcomes.

**Methods:**

This study included 179 lipid species (*n* = 7174) and 91 circulating inflammatory factors (*n* = 14 824) as exposure variables, with preterm birth data from the FinnGen database (*n* = 186 212) serving as the outcome variable. We applied a two‐sample Mendelian randomization approach and mediation analysis techniques to identify specific biomarkers causally associated with preterm birth and to investigate the relationships between them from a genetic perspective.

**Results:**

In this study, we identified 11 lipid metabolites that show a significant causal association with preterm birth, of which eight are positively correlated and three are negatively correlated. Additionally, we identified 18 circulating inflammatory factors with a significant causal relationship to preterm birth, with 10 showing a negative correlation and eight a positive correlation. Adenosine deaminase (ADA) served as a crucial protective factor against preterm birth (*P* = 0.006, 95% confidence interval [CI] 0.90–0.98) and mediated the causal relationships between various lipid metabolites and preterm birth.

**Conclusion:**

This Mendelian randomization study identifies dysregulated lipid metabolism and inflammatory pathways influencing preterm birth. It identifies dysregulated lipid metabolism and inflammatory pathways influencing preterm birth. ADA is a significant protective factor against preterm birth. Specific phosphatidylcholine subtypes exert their protective effects against preterm birth through an ADA‐dependent pathway. The mediation effect was −0.005 (95% CI –0.01 to −0.001). This finding not only deepens our understanding of the inflammatory origins of preterm birth but also provides direct evidence for the development of precision prevention strategies based on the regulation of the lipid‐inflammation axis.

## 1 INTRODUCTION

Preterm birth, which is defined as delivery before 37 weeks of pregnancy, affects approximately 5%–18% of pregnancies.^1^, ^2^ It represents a leading cause of neonatal morbidity and mortality and the second most frequent cause of death among children under 5 years of age.^3^ Preterm birth has long‐lasting effects on child growth and development, which may persist into adulthood,^4^, ^5^ thereby imposing significant social and familial burdens. The etiology of preterm birth is multifactorial and complex, involving diverse pathologic processes with as yet unclear mechanisms. At present, no effective methods exist for predicting or treating preterm birth.^6^ Therefore, investigating the mechanisms underlying preterm birth and identifying potential therapeutic targets for its prevention and treatment hold significant clinical relevance.

Lipid metabolites play a crucial role in the occurrence and development of preterm birth.^7^ Studies have demonstrated a significant causal association between lipid metabolism disorders and preterm birth. Changes in lipid metabolites may influence the risk of preterm birth through various mechanisms, including oxidative stress, immune modulation, and inflammatory responses.^8^, ^9^ Significant dysregulation of lipid metabolism has been observed in patients with preterm birth, particularly concerning lipids and fatty acids. These metabolic disorders may increase the risk of preterm birth by affecting placental function, fetal development, and maternal health. Lipid metabolism disorders can lead to increased oxidative stress, which in turn triggers inflammatory responses and ultimately results in preterm birth.^10^, ^11^, ^12^ However, the causal relationship between preterm birth and lipid metabolites has not been investigated. Consequently, exploring the causal relationship between specific lipid metabolism, and preterm birth is essential to advancing our understanding of the underlying mechanisms.

Meanwhile, parturition is widely acknowledged as an inflammatory event, and preterm birth is regarded as a premature initiation of this process.^13^ Parturition encompasses three primary pathways: rupture of the fetal membranes, cervical dilation, and uterine contractions.^14^ The regulation of inflammatory cytokines is essential for the execution of these processes. Previous studies have highlighted that a variety of inflammatory factors, including members of the interleukin and tumor necrosis factor families, as well as chemokines and interferons, play critical roles in this process.^15^, ^16^, ^17^, ^18^ However, the number of inflammatory cytokines implicated in regulating this process is significantly greater than those currently identified. Alterations in the levels and functional activity of these cytokines may contribute to the onset of preterm birth. Moreover, the causal relationships and underlying mechanisms connecting these inflammatory cytokines to preterm birth remain poorly understood, underscoring the clinical importance of further exploring the causal association between preterm birth and circulating inflammatory factors.

Mendelian randomization (MR) employs single nucleotide polymorphisms (SNPs) as instruments to evaluate causal associations between exposures and outcomes.^19^ In this study, we applied both two‐sample MR and mediation MR approaches to rigorously investigate how lipid metabolites and circulating inflammatory factors contribute to the occurrence of preterm birth and their interactions. We anticipate that the findings from this research will provide valuable insights and offer novel perspectives for the prevention and management of preterm birth.

## 2 MATERIALS AND METHODS

### 2.1 Data sources

The data for this MR study were obtained from the publicly available dataset of the FinnGen Consortium of Genomics and Health. Specifically, we analyzed the finngen_R10_O15_PRETERM dataset, which includes a case group comprising 9402 individuals and a control group consisting of 176 810 European participants.

The lipidomic data were derived from the study titled “Genome‐wide association analysis of the plasma lipidome identifies 495 genetic associations,” published in *Nature Communications* in 2023.^20^ This study conducted a genome‐wide association study in 7174 Finnish participants, investigating 179 lipids. Using shotgun lipidomics, the study identified 179 lipids across 13 lipid categories, covering four major lipid classes: triglycerides, glycerophospholipids, sphingolipids, and sterols.

The data for circulating inflammatory factors were sourced from a study published in *Nature Immunology* titled “Genetics of circulating inflammatory proteins identifies drivers of immune‐mediated disease risk and therapeutic targets,” in 2023.^21^ In this study, the authors performed a genome‐wide protein quantitative trait locus mapping of 91 plasma proteins across 11 cohorts, involving a total of 14 824 European participants using the Olink Target platform.

All data required for this study were sourced from publicly accessible summary statistics of genome‐wide association studies. No new data were collected for this study, nor was additional ethical approval required.

### 2.2 Mendelian randomization analysis method

In this MR study, we employed the following criteria for the selection of appropriate instrumental variables.^22^ (1) We adopted a threshold of *P* less than 1 × 10^−5^ for selecting SNPs. (2) To minimize the effects of linkage disequilibrium, we performed a clustering analysis with a threshold of *r* ^2^ less than 0.1 and a window size of 500 kb. (3) To ensure the strength of the instrumental variables, we evaluated their relevance using the F‐statistic, excluding any SNPs with an F‐statistic below 10 to mitigate weak instrument bias.^23^ The formula for the *F*‐statistic is *F* = (*R* ^2^ × [*n* − *k* − 1])/(*k* × [1 − *R* ^2^]), where *R* ^2^ = 2 × β^2^ × EAF × (1 − EAF)/(2 × β^2^ × EAF × [1 − EAF] + 2 × SE^2^ × *n* × EAF × [1 − EAF]), with *n* representing the sample size, *k* denoting the number of instruments and EAF (Exposure Allele Frequency) being the frequency of the allele associated with the exposure variable in the population. (4) SNPs located within palindromic sequences were excluded to prevent potential analysis biases. (5) Steiger filtering was applied to ensure the validity of the instrumental variables. This rigorous selection process ensures the identification of the most appropriate instruments, thereby providing a robust foundation for subsequent causal inference.

Subsequently, we used five methods to evaluate causality: inverse variance weighting (IVW), MR‐Egger, weighted median, simple mode, and weighted mode. The IVW method served as the primary analytical tool, while the other four methods were employed as supplementary analyses to enhance the robustness of the results. A value of *P* of less than 0.05 was set as the threshold for determining the significance of causal relationships. Additionally, we conducted sensitivity analyses using Cochran Q test, Egger intercept test, MR‐PRESSO, and the leave‐one‐out method to further assess potential issues of heterogeneity and pleiotropy. A *P* value greater than 0.05 indicated the absence of these issues. All analyses were performed using the TwoSampleMR and MRPRESSO packages in R software (version 4.3.3).^24^

### 2.3 Mediation Mendelian randomization analysis

In this study, we employed a two‐sample MR approach to investigate the roles of 179 lipids and 91 circulating inflammatory factors in preterm birth, and further explored the mediating effects of circulating inflammatory factors in the genetic causal relationship between lipids and preterm birth. Initially, we identified lipids and circulating inflammatory factors that were positively associated with preterm birth through two‐sample MR analysis. Subsequently, we examined the correlations between these positive lipids and positive circulating inflammatory factors to identify potential mediators. The methodology for two‐sample MR analysis has been previously described.

For the mediation analysis, we first assessed the impact of lipids on preterm birth and calculated the total effect, denoted as β. This total effect can be decomposed into two components: the “direct” effect, which represents the impact of the primary exposure on the outcome, independent of the mediator included in the model, indicated by an arrow from lipids to preterm birth and labeled as β; and the “indirect” effect, which represents the effect of the primary exposure on the outcome via the mediator. The mediating effect was calculated using the product‐of‐coefficients method (β1 × β2), where β1 represents the causal effect of positive lipids on positive circulating inflammatory factors, and β2 represents the causal effect of positive circulating inflammatory factors on preterm birth. The proportion of the mediating effect was calculated as the ratio of the “indirect effect” to the “total effect.”

### 2.4 Research design

**FIGURE 1:**
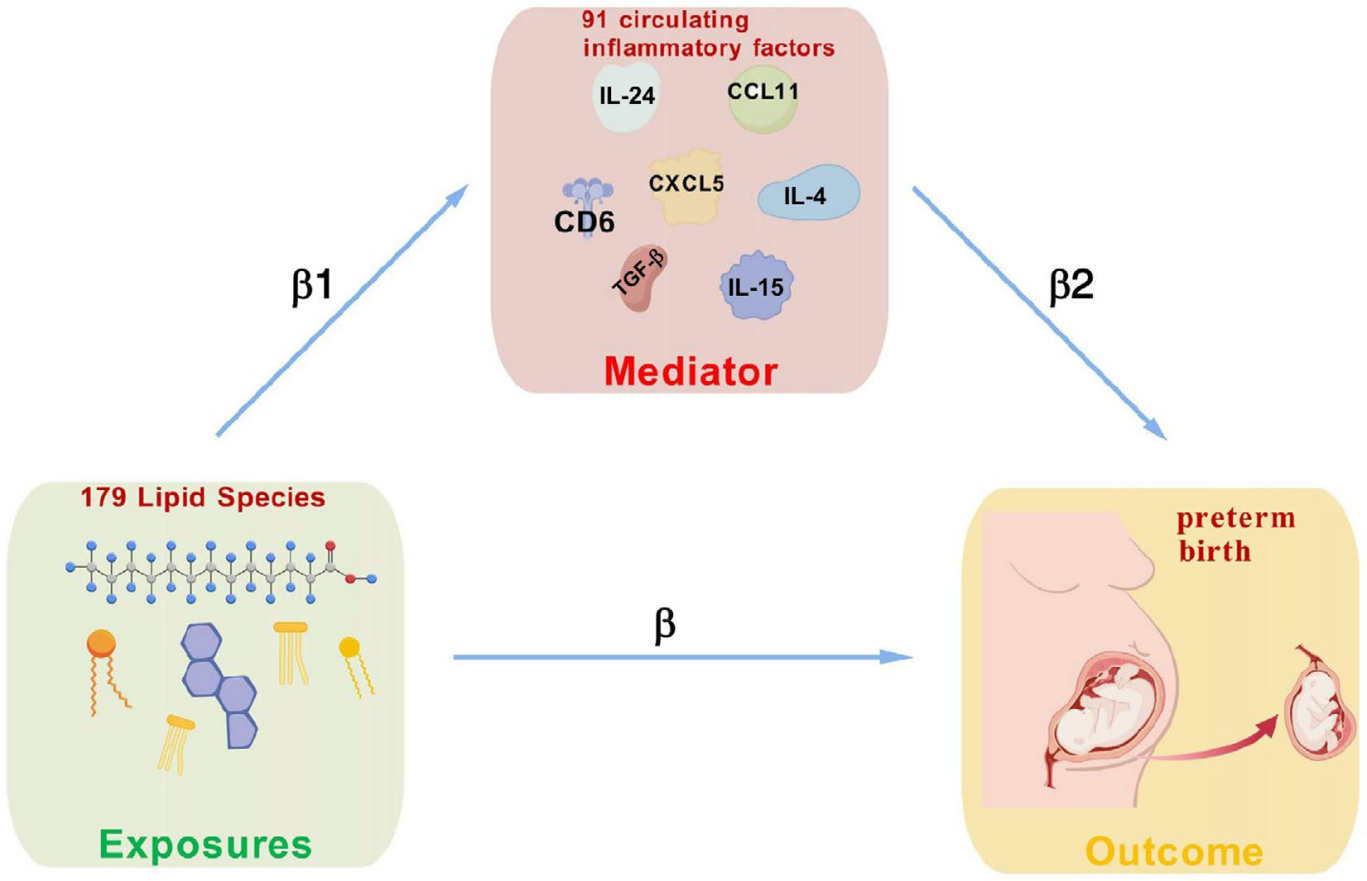
β, Total effect size of the lipid species on preterm birth; β1, Causal effect of the circulating inflammatory factors on the lipidome; β2, Causal effect of the circulating inflammatory factors on pregnancy outcomes.

The specific analytical process is as described elsewhere^25^ (Figure 1).

## 3 RESULTS

### 3.1 Mendelian randomization analysis results

**FIGURE 2:**
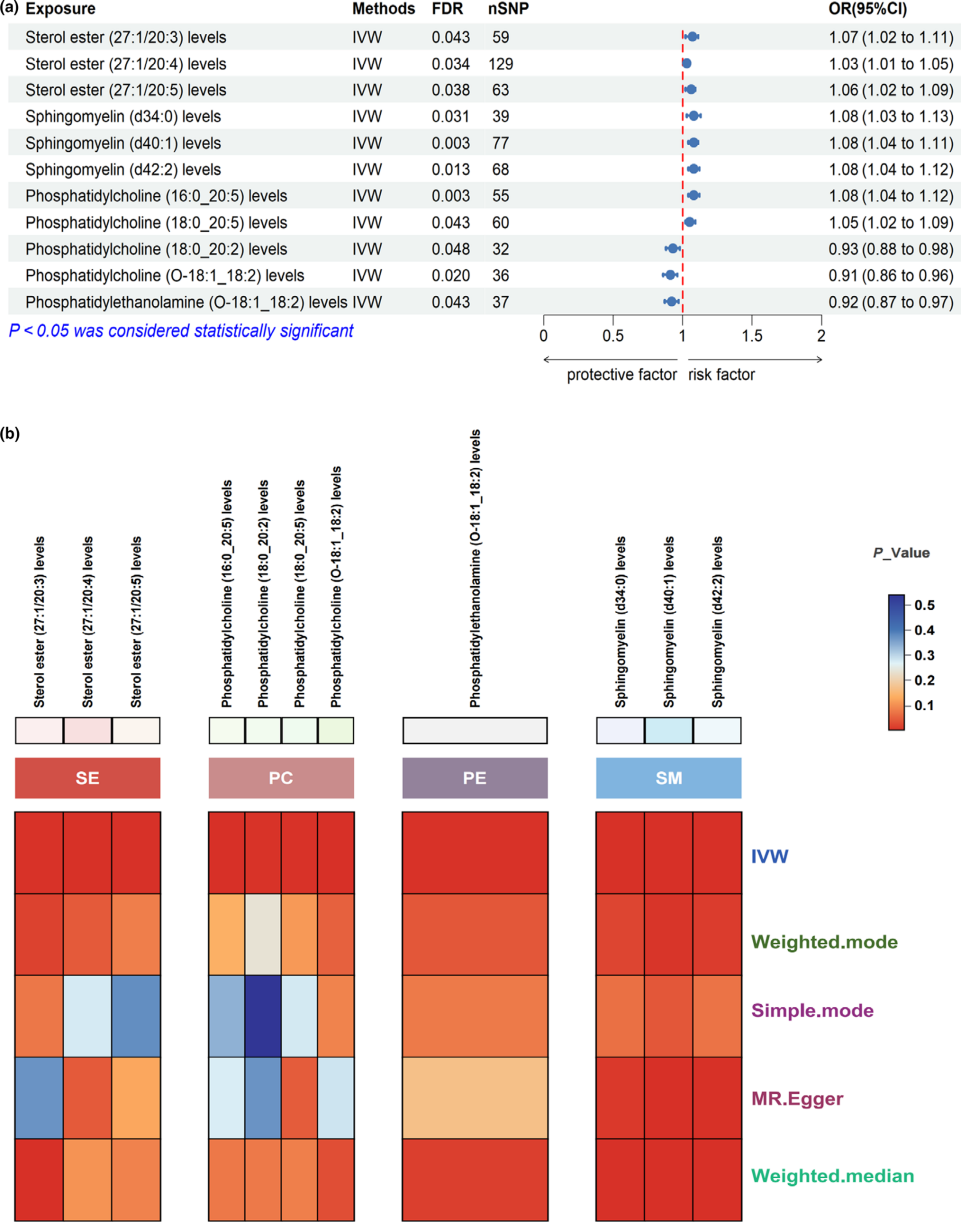
(a) Forest plot illustrating the significant causal estimates between the lipidome and preterm birth. CI, confidence interval; FDR, false discovery rate; IVW, inverse variance weighting; OR, odds ratio; SNPs, single nucleotide polymorphisms. (b) Heatmap depicting the positive lipid groups. PC, phosphatidylcholines; PE, phosphatidylethanolamines; SE, sterol esters; SM, sphingomyelins.

In the two‐sample MR analysis, we used SNPs with *P* values less than 1 × 10^−5^ (see Data S1) as instrumental variables and selected 179 lipid metabolites for analysis. The significance criterion for causal associations was set at a false discovery rate of less than 0.05 using the IVW method. Our analysis identified 11 lipid metabolites with significant causal relationships with preterm birth (Figure 2a), including three protective factors: phosphatidylcholine (O‐18:1_18:2), phosphatidylethanolamine (O‐18:1_18:2), and phosphatidylcholine (18:0_20:2). Additionally, eight lipid metabolites were identified as risk factors: cholesteryl ester (27:1/20:4), phosphatidylcholine (18:0_20:5), cholesteryl ester (27:1/20:5), cholesteryl ester (27:1/20:3), sphingomyelin (d40:1), sphingomyelin (d42:2), sphingomyelin (d34:0), and phosphatidylcholine (16:0_20:5). We found that the positive exposures primarily fell into four major categories. The role of phosphatidylcholines was relatively complex, with some acting as risk factors and others as protective factors, whereas sterols and sphingolipids were consistently identified as risk factors, and phosphatidylethanolamines were found to be protective factors. We further validated these findings using several other MR analysis methods, which showed consistent trends, although some did not reach statistical significance (Figure 2b).

**FIGURE 3:**
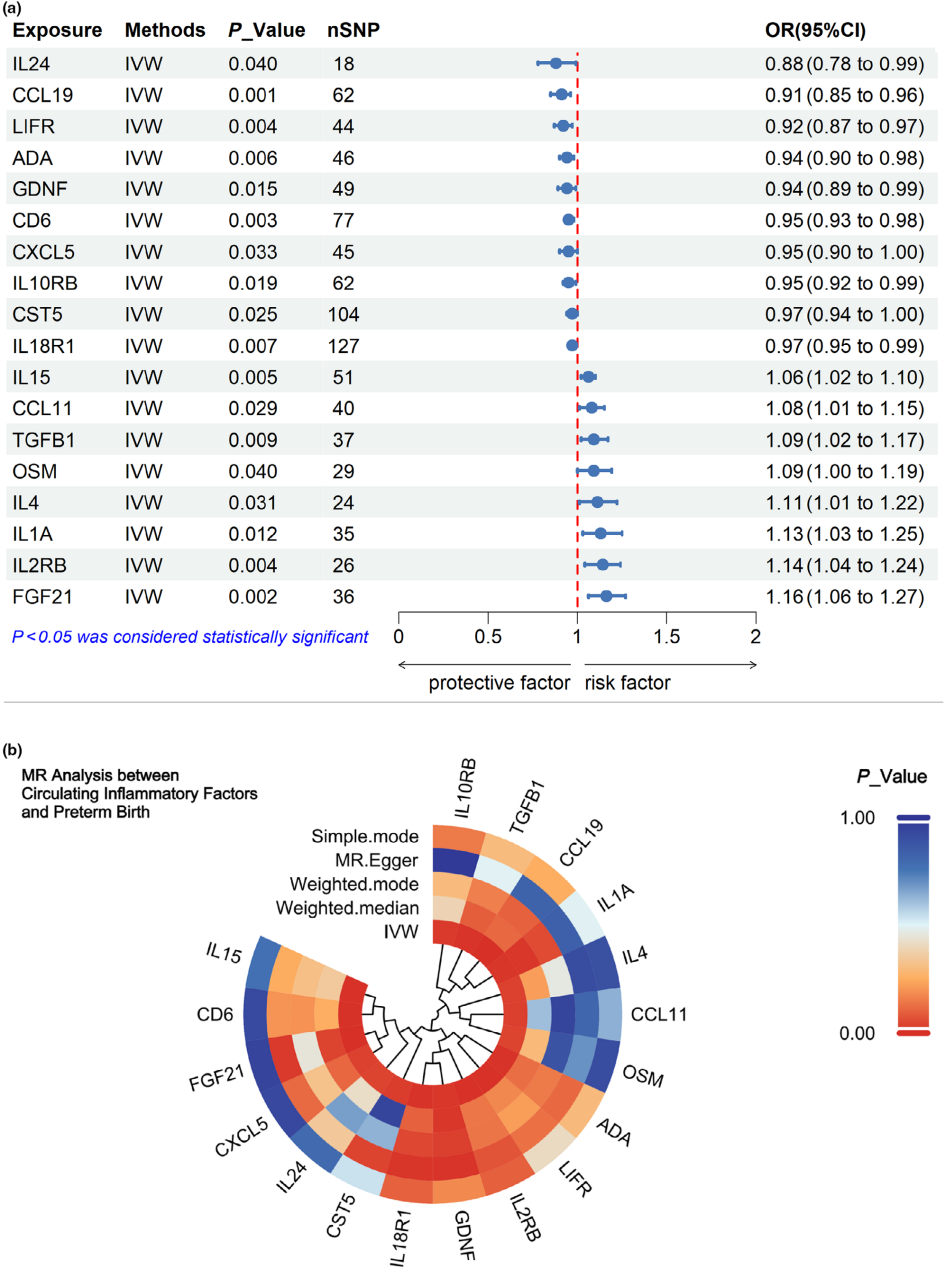
(a) Forest plot of the significant causal estimates between circulating inflammatory factors and preterm birth. CI, confidence interval; IVW, inverse variance weighted; OR, odds ratio; SNPs, single nucleotide polymorphisms. (b) Circular heatmap of Mendelian randomization (MR) analysis between circulating inflammatory factors and preterm birth.

To maximize the identification of potential causal relationships between circulating inflammatory factors and preterm birth, we relaxed the constraints on SNPs by setting a significance threshold of *P* less than 1 × 10^−5^ (Data S1) for SNPs used as instrumental variables in the two‐sample MR analysis. To determine the significance of causal associations, we applied a *P* value threshold of less than 0.05 using the IVW method. Our analysis identified 10 circulating inflammatory factors as protective factors against the occurrence and progression of preterm birth, including IL‐24, LIFR, GDNF, ADA, CXCL5, IL‐10RB, CD6, IL‐18R1, CST5, and CCL19. Additionally, eight proteins were identified as risk factors, including TGF‐β1, CCL11, OSM, IL‐15, IL‐4, IL‐2RB, FGF21, and IL‐1A (Figure 3a). Other MR methods, including the simple mode, weighted mode, and weighted median analysis, generally yielded results consistent with the IVW method, although some did not reach statistical significance (Figure 3b).

### 3.2 Sensitivity analysis results

Subsequently, we conducted a series of sensitivity analyses on the 18 circulating inflammatory factors and 11 lipid metabolites identified above to further evaluate the robustness of our findings. The criteria for assessing heterogeneity and horizontal pleiotropy were as follows: Cochran Q *P* less than 0.05 for heterogeneity, MR‐PRESSO Global_test_p greater than 0.05 for the global test, and Egger intercept *P* greater than 0.05 for horizontal pleiotropy. Among the 11 positive lipid metabolites, no significant heterogeneity or horizontal pleiotropy was detected (Table 1). However, the MR‐PRESSO Global_test_p values for sphingomyelin (d40:1) and sphingomyelin (d42:2) were both less than 0.05, indicating the presence of horizontal pleiotropy (*P* < 0.05). For the 18 positive circulating inflammatory factors, heterogeneity was observed in FGF21, IL‐1A, and CCL19 (Cochran Q *P* < 0.05). The MR‐PRESSO analysis revealed horizontal pleiotropy in CCL19, CST5, FGF21, and IL‐1A (Global_test_p < 0.05), while the Egger intercept method indicated no horizontal pleiotropy for any of these factors (*P* > 0.05) (Table 1). The Leave‐One‐Out analysis confirmed the robustness of our results (see Data S2). Additionally, we performed reverse MR analysis and found no evidence of reverse causal associations between the 11 positive lipid metabolites and preterm birth, nor between the 18 positive circulating inflammatory factors and preterm birth (see Data S1).

**TABLE 1:** Sensitivity analysis results of lipidome and preterm birth.

Exposure	Method	Cochran's Q		MR‐PRESSO	Egger intercept	Reverse MR (IVW)	
		Q	*P*	Global test_p	*P*	*P*	OR (95% CI)
Sterol ester (27:1/20:3) levels	IVW	71.504	0.110	0.118	0.803	0.648	0.98 (0.90–1.07)
Sterol ester (27:1/20:4) levels	IVW	134.967	0.319	0.354	0.747	0.483	1.03 (0.95–1.12)
Sterol ester (27:1/20:5) levels	IVW	61.119	0.508	0.540	0.967	0.745	0.99 (0.91–1.07)
Phosphatidylcholine (16:0_20:5) levels	IVW	41.708	0.889	0.895	0.318	0.275	0.96 (0.88–1.04)
Phosphatidylcholine (18:0_20:2) levels	IVW	29.536	0.541	0.599	0.760	0.251	0.95 (0.86–1.04)
Phosphatidylcholine (18:0_20:5) levels	IVW	45.840	0.895	0.897	0.589	0.380	0.96 (0.89–1.05)
Phosphatidylcholine (O‐18:1_18:2) levels	IVW	42.679	0.174	0.201	0.860	0.571	1.02 (0.94–1.12)
Phosphatidylethanolamine (O‐18:1_18:2) levels	IVW	27.152	0.856	0.886	0.840	0.290	1.06 (0.95–1.18)
Sphingomyelin (d34:0) levels	IVW	39.439	0.405	0.434	0.273	0.874	0.99 (0.91–1.08)
Sphingomyelin (d40:1) levels	IVW	75.195	0.505	0.546	0.014	0.081	0.93 (0.86–1.01)
Sphingomyelin (d42:2) levels	IVW	72.380	0.305	0.331	0.042	0.535	0.97 (0.90–1.06)

Abbreviations: CI, confidence interval; IVW, inverse variance weighted; MR, Mendelian randomization; OR, odds ratio.

### 3.3 Mediation analysis

Subsequently, we conducted mediation analyses to identify circulating inflammatory factors that may act as mediators (Table 2). Initially, we examined the causal associations between the 11 positively selected lipids and 18 positively selected circulating inflammatory factors (see Data S1) and performed mediation analyses (Table 3). Only molecular pairs demonstrating statistically significant causal associations (IVW *P* < 0.05) that were consistent with a unidirectional causal hypothesis (lipid metabolites to inflammatory factors) were retained for subsequent analyses. Notably, adenosine deaminase (ADA) serves as a mediator for various lipids and warrants focused attention, particularly regarding its role in mediating phosphatidylcholine (18:0/20:2). Our study indicates that both ADA and phosphatidylcholine (18:0/20:2) act as protective factors against preterm birth.

**TABLE 2:** Sensitivity analysis for circulating inflammatory factors and preterm birth.

Exposure	Method	Cochran's Q		MR‐PRESSO	Egger intercept	Reverse MR (IVW)	
		Q	*P*	Global test_p	*P*	*P*	OR (95% CI)
ADA	IVW	55.269	0.140	0.164	0.965	0.724	1.01 (0.96–1.06)
CCL11	IVW	44.663	0.246	0.244	0.157	0.316	0.97 (0.92–1.03)
CCL19	IVW	92.854	0.005	0.004	0.054	0.883	0.96 (0.90–1.03)
CD6	IVW	68.567	0.715	0.700	0.589	0.261	0.98 (0.93–1.03)
CST5	IVW	119.637	0.125	0.043	0.328	0.758	1.01 (0.96–1.06)
CXCL5	IVW	40.482	0.623	0.672	0.673	0.440	1.03 (0.96–1.10)
FGF21	IVW	50.249	0.046	0.009	0.218	0.905	0.99 (0.94–1.05)
GDNF	IVW	42.891	0.682	0.678	0.086	0.710	0.99 (0.94–1.04)
IL10RB	IVW	44.081	0.950	0.954	0.082	0.400	1.00 (0.94–1.06)
IL15	IVW	60.509	0.147	0.167	0.644	0.843	0.97 (0.92–1.03)
IL18R1	IVW	113.080	0.788	0.813	0.271	0.658	1.00 (0.94–1.06)
IL1A	IVW	59.034	0.005	0.005	0.177	0.659	0.99 (0.94–1.04)
IL24	IVW	19.908	0.279	0.296	0.064	0.978	0.99 (0.93–1.04)
IL2RB	IVW	27.025	0.355	0.393	0.504	0.372	0.99 (0.93–1.06)
IL4	IVW	22.208	0.508	0.537	0.359	0.891	1.01 (0.96–1.06)
TGFB1	IVW	35.758	0.480	0.508	0.439	0.600	0.97 (0.92–1.03)
LIFR	IVW	42.612	0.488	0.525	0.822	0.601	0.96 (0.90–1.03)
OSM	IVW	18.861	0.902	0.903	0.556	0.809	0.98 (0.93–1.03)

Abbreviations: CI, confidence interval; IVW, inverse variance weighted; MR, Mendelian randomization; OR, odds ratio.

**TABLE 3:** Causal effects of lipid metabolites on preterm birth mediated by circulating inflammatory factors.^a^

Exposure	Mediator	Outcome	Mediation effect	Total effect	Indirect proportion	Mediation effect (95% CI)
Sterol ester (27:1/20:3) levels	IL18R1	preterm	0.002	0.064	0.025	0.000–0.004
	IL24	preterm	−0.006	0.064	−0.099	−0.016 to 0.001
	IL4	preterm	0.004	0.064	0.066	−0.001 to 0.011
Sterol ester (27:1/20:4) levels	ADA	preterm	0.003	0.033	0.089	0.001–0.006
	IL18R1	preterm	0.002	0.033	0.052	0.001–0.003
	TGFB1	preterm	−0.004	0.033	−0.121	−0.008 to −0.001
Sterol ester (27:1/20:5) levels	ADA	preterm	0.004	0.055	0.064	0.001–0.007
	CD6	preterm	0.003	0.055	0.061	0.001–0.007
	CXCL5	preterm	0.003	0.055	0.059	0.001–0.007
	IL15	preterm	−0.002	0.055	−0.038	−0.005 to −0.001
	IL18R1	preterm	0.003	0.055	0.047	0.001–0.005
	IL4	preterm	−0.004	0.055	−0.065	−0.010 to 0.001
	TGFB1	preterm	−0.005	0.055	−0.090	−0.010 to −0.001
Phosphatidylcholine (16:0_20:5) levels	ADA	preterm	0.003	0.080	0.035	0.001–0.007
	CD6	preterm	0.003	0.080	0.042	0.001–0.007
	CXCL5	preterm	0.003	0.080	0.041	0.001–0.007
	IL18R1	preterm	0.002	0.080	0.031	0.001 − 0.005
	TGFB1	preterm	–0.005	0.080	−0.056	−0.010 to −0.001
Phosphatidylcholine (18:0_20:2) levels	ADA	preterm	−0.005	−0.074	0.061	−0.009 to −0.001
	CXCL5	preterm	−0.004	−0.074	0.058	−0.010 to −0.001
	TGFB1	preterm	0.007	−0.074	−0.089	0.001–0.014
Phosphatidylcholine (18:0_20:5) levels	ADA	preterm	0.003	0.053	0.058	0.001–0.006
	CD6	preterm	0.003	0.053	0.050	0.001–0.006
	CXCL5	preterm	0.002	0.053	0.044	0.001–0.006
	IL15	preterm	−0.002	0.053	−0.037	−0.005 to −0.001
	IL4	preterm	−0.004	0.053	−0.075	−0.010 to 0.001
Phosphatidylethanolamine (O‐18:1_18:2) levels	IL18R1	preterm	−0.002	−0.085	0.021	−0.004 to −0.001
Sphingomyelin (d34:0) levels	CCL11	preterm	−0.003	0.077	−0.041	−0.008 to 0.001
	IL4	preterm	−0.007	0.077	−0.085	−0.016 to −0.001
Sphingomyelin (d40:1) levels	CD6	preterm	0.002	0.074	0.025	0.001–0.004
	FGF21	preterm	−0.005	0.074	−0.071	−0.012 to −0.001

Abbreviation: CI, confidence interval.

^a^ Total effect denotes the influence of lipidome on preterm birth, ascertained via the inverse variance weighted (IVW) method. Indirect effect signifies the influence of lipidomes on preterm birth mediated through circulating inflammatory factors, calculated using the Delta method. The Indirect proportion represents the ratio of the total effect that is mediated by circulating inflammatory factors, Indirect proportion = Indirect effect/Total effect. A mediation effect is deemed significant if its 95% CI does not encompass zero.

## 4 DISCUSSION

In our research findings, we first identified 11 lipids and 18 circulating inflammatory proteins that have a causal relationship with preterm birth. These 11 lipids are primarily categorized into four major classes: phosphatidylcholines, phosphatidylethanolamines, sterols, and sphingolipids. Sterol esters and sphingolipids are both associated with an increased risk of preterm birth, whereas phosphatidylethanolamine appears to exert a protective effect.

Sterol esters are the primary storage form of intracellular cholesterol and play a key role in regulating the systemic distribution of cholesterol. During pregnancy, the placenta requires a large amount of cholesterol to synthesize steroid hormones (such as progesterone) essential for fetal and placental development.^26^ Previous studies have suggested that maternal hypercholesterolemia^27^ is associated with an increased risk of preterm birth, potentially as the result of placental dysfunction and inflammatory responses.^28^ Sphingomyelin,^29^ an essential component of the cellular membrane, when altered, may disrupt cellular structure and function, thereby contributing to the occurrence of preterm birth.

Phosphatidylethanolamine is a crucial component of the cell membrane.^30^ Trophoblast cells of the placenta require intact and functional membranes to support their invasive, migratory, and differentiative capacities^31^; thus, phosphatidylethanolamine can directly influence placental function. Second, phosphatidylethanolamine is closely related to mitochondrial function.^32^ Placental mitochondrial dysfunction leads to increased oxidative stress, which impairs fetal nutrient supply and development, thereby increasing the risk of preterm birth.^33^ Additionally, mitochondrial dysfunction in immune cells such as macrophages and dendritic cells can impair their immunoregulatory capacity,^34^ potentially causing immune dysregulation, which contributes to the occurrence of preterm birth. Therefore, phosphatidylethanolamine may affect pregnancy immunity through the modulation of mitochondrial function. Phosphatidylcholine,^35^ an important membrane phospholipid, displays a complex role in our study. Some species appear protective, whereas others are associated with increased risk. This duality likely arises from their influence on placental function and immune regulation, although the precise mechanisms warrant further investigation.

Preterm birth is widely recognized as an inflammatory event.^2^ The prevailing consensus is that pro‐inflammatory cytokines and proteins promote preterm birth, whereas anti‐inflammatory cytokines and proteins inhibit its occurrence. Consistent with previous studies, our findings demonstrate a positive correlation between the pro‐inflammatory factors CCL11^36^ and IL‐1A^37^ and preterm birth, whereas the anti‐inflammatory factors GDNF^38^ and IL‐10RB^39^ exhibit a negative correlation. Notably, although previous research has identified CXCL5^40^ and IL‐18R1^41^ as pro‐inflammatory mediators, our study reveals a protective association of these factors against preterm birth. Conversely, TGF‐β1^42^ and IL‐4,^43^ which are known to suppress inflammation, showed a positive correlation with preterm birth, a seemingly paradoxical observation that warrants further validation to exclude confounding effects. Moreover, several cytokines exhibit more complex, immunoregulatory roles, including CST5,^44^ IL‐24,^45^ FGF21,^46^ LIFR,^47^ CD6,^48^ IL‐2RB,^49^ CCL19,^50^ ADA,^51^ OSM,^52^ and IL‐15.^53^ In our cohort, FGF21, IL‐2RB, and OSM were positively correlated with preterm birth, whereas LIFR, CD6, IL‐15, IL‐24, CCL19, CST5, and ADA showed negative correlations. These molecules may serve as critical targets for future prediction and therapeutic intervention of preterm birth. Through mediation analysis, we identified that ADA serves as a mediator in the associations between multiple lipid species and preterm birth, particularly mediating the protective effect of phosphatidylcholine (18:0_20:2) levels against preterm birth. This finding highlights ADA as a critical focus for further investigation. ADA is known to regulate adenosine metabolism^54^ and is closely involved in the functions of various immune cells,^51^ thereby maintaining immune homeostasis. Based on these roles, ADA may protect the organism from the detrimental effects of preterm birth. The underlying mechanisms warrant in‐depth exploration in future studies.

Recent advances have demonstrated that pre‐pregnancy obesity substantially increases the risk of preterm birth,^55^ potentially through interactions between lipid metabolism and chronic inflammatory responses. However, the interplay between lipids and inflammation is highly complex. Dysregulation of lipid metabolism can initiate inflammatory processes, and conversely, inflammation can modulate lipid metabolism. By investigating specific interactions between lipids and inflammatory factors, our study identifies phosphatidylcholine as a potential agent that reduces preterm birth risk by modulating ADA activity. This pathway offers novel insights into the mechanisms underlying preterm birth. Furthermore, this finding refines the “metabolic‐immune axis” theoretical framework and provides critical molecular targets for developing lipid metabolism‐based preventive strategies and precision medicine interventions against preterm birth.

Although this study provides valuable insights into the relationships between the lipidome, circulating inflammatory factors, and preterm birth, several limitations may affect the interpretation of our findings. First, we used publicly available data from genome‐wide association studies to analyze the lipidome and inflammatory proteins. Although these datasets encompass a wide range of lipids and inflammatory markers, they may not fully capture the complete spectrum of lipid and immune variation, potentially leading to an incomplete characterization of exposure traits. Second, the identified lipid and inflammatory factors were based on genome‐wide association study summary statistics, with no access to individual‐level data. This limitation restricted our ability to explore dose–response relationships and assess the continuous impact of lipids and circulating inflammatory factors on the risk of preterm birth. Finally, the study population primarily consisted of individuals of European ancestry, which limits the generalizability of our findings. Further investigations in other populations, particularly those with diverse ethnic or geographic backgrounds, are warranted.

## AUTHOR CONTRIBUTIONS

SX made contributions across all aspects of the research, including the conceptualization, design, data collection and analysis, and manuscript writing and revision. CW approved the final manuscript for submission.

## CONFLICT OF INTEREST STATEMENT

The authors have no conflicts of interest.

## Supporting information


Data S1.

Data S2.

## Data Availability

The original contributions of this study are detailed in the text and supporting information. For further information, please contact the corresponding author directly.
