# Supplementary material for: Lipid metabolism and inflammation as key drivers in preterm birth: A comprehensive analysis
Source: Int J Gynaecol Obstet. 2025 Jun 7;171(3):1212–22. doi: 10.1002/ijgo.70285 (PMC12640176; doi:10.1002/ijgo.70285)
Supplement: Supplementary file 1 — Data S1. [file IJGO-171-1212-s001.pdf]

A

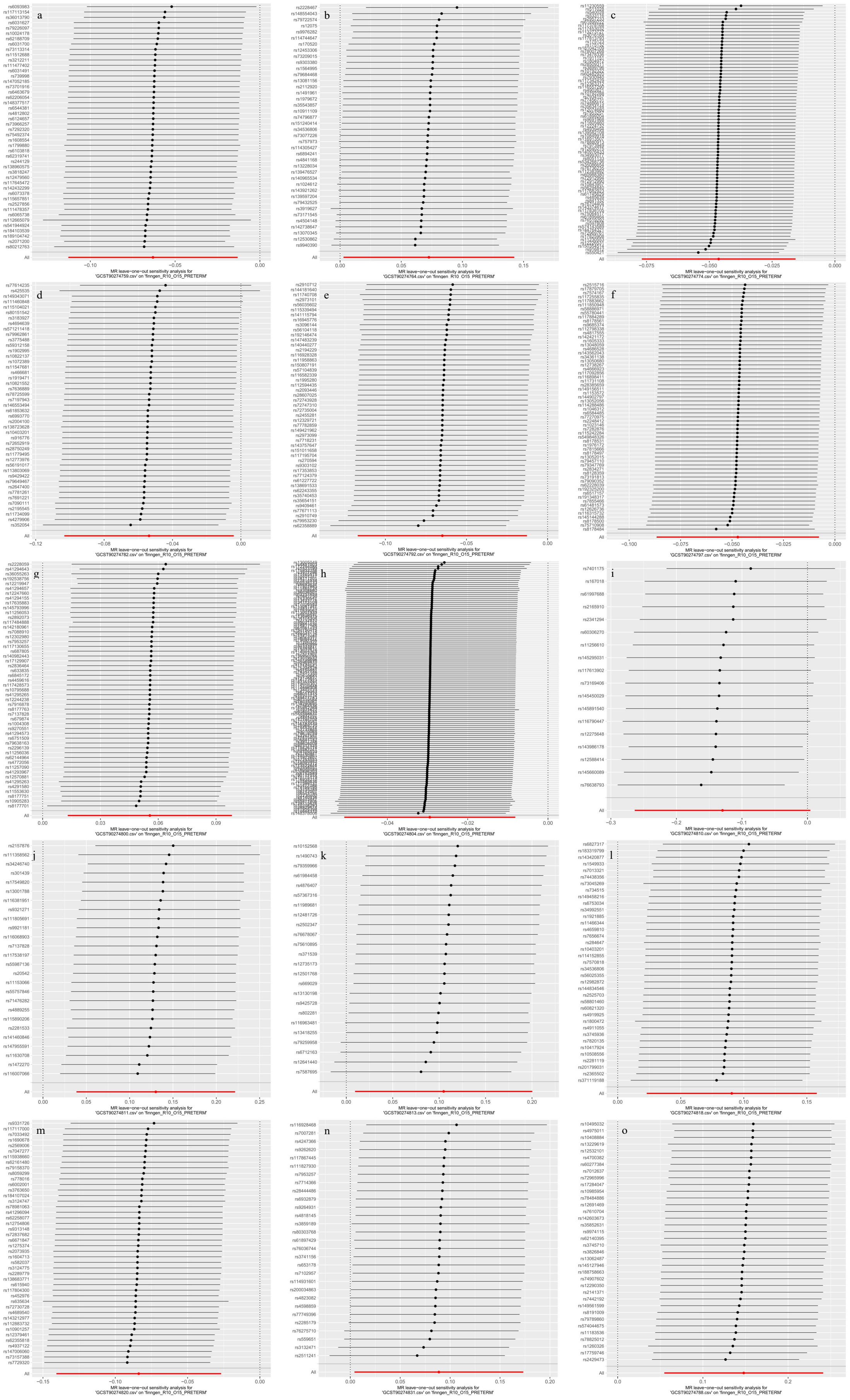
a
MR leave-one-out sensitivity analysis for 'GCST90274759.csv' on 'finngen_R10_O15_PRETERM'
b
MR leave-one-out sensitivity analysis for 'GCST90274764.csv' on 'finngen_R10_O15_PRETERM'
c
MR leave-one-out sensitivity analysis for 'GCST90274774.csv' on 'finngen_R10_O15_PRETERM'
d
MR leave-one-out sensitivity analysis for 'GCST90274782.csv' on 'finngen_R10_O15_PRETERM'
e
MR leave-one-out sensitivity analysis for 'GCST90274792.csv' on 'finngen_R10_O15_PRETERM'
f
MR leave-one-out sensitivity analysis for 'GCST90274797.csv' on 'finngen_R10_O15_PRETERM'
g
MR leave-one-out sensitivity analysis for 'GCST90274800.csv' on 'finngen_R10_O15_PRETERM'
h
MR leave-one-out sensitivity analysis for 'GCST90274804.csv' on 'finngen_R10_O15_PRETERM'
i
MR leave-one-out sensitivity analysis for 'GCST90274810.csv' on 'finngen_R10_O15_PRETERM'
j
MR leave-one-out sensitivity analysis for 'GCST90274811.csv' on 'finngen_R10_O15_PRETERM'
k
MR leave-one-out sensitivity analysis for 'GCST90274813.csv' on 'finngen_R10_O15_PRETERM'
l
MR leave-one-out sensitivity analysis for 'GCST90274818.csv' on 'finngen_R10_O15_PRETERM'
m
MR leave-one-out sensitivity analysis for 'GCST90274820.csv' on 'finngen_R10_O15_PRETERM'
n
MR leave-one-out sensitivity analysis for 'GCST90274831.csv' on 'finngen_R10_O15_PRETERM'
o
MR leave-one-out sensitivity analysis for 'GCST90274788.csv' on 'finngen_R10_O15_PRETERM'

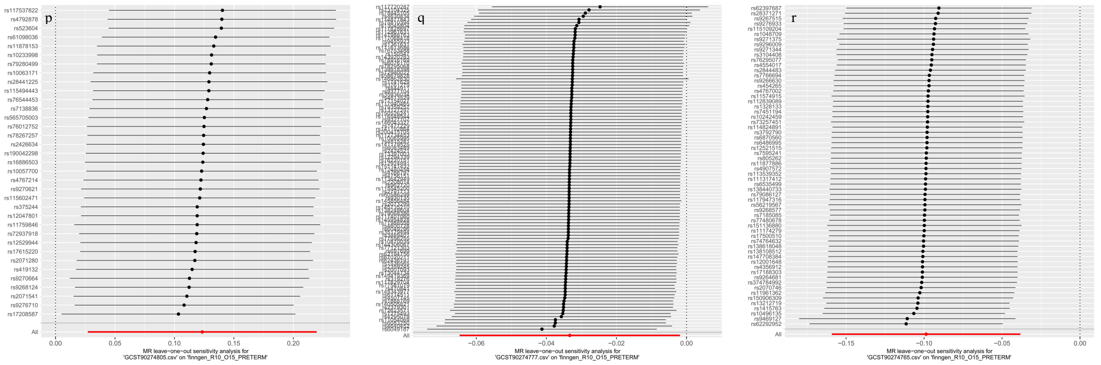
p
MR leave-one-out sensitivity analysis for
'GCST90274805.csv' on 'finngen_R10_O15_PRETERM'
q
MR leave-one-out sensitivity analysis for
'GCST90274777.csv' on 'finngen_R10_O15_PRETERM'
r
MR leave-one-out sensitivity analysis for
'GCST90274765.csv' on 'finngen_R10_O15_PRETERM'

B

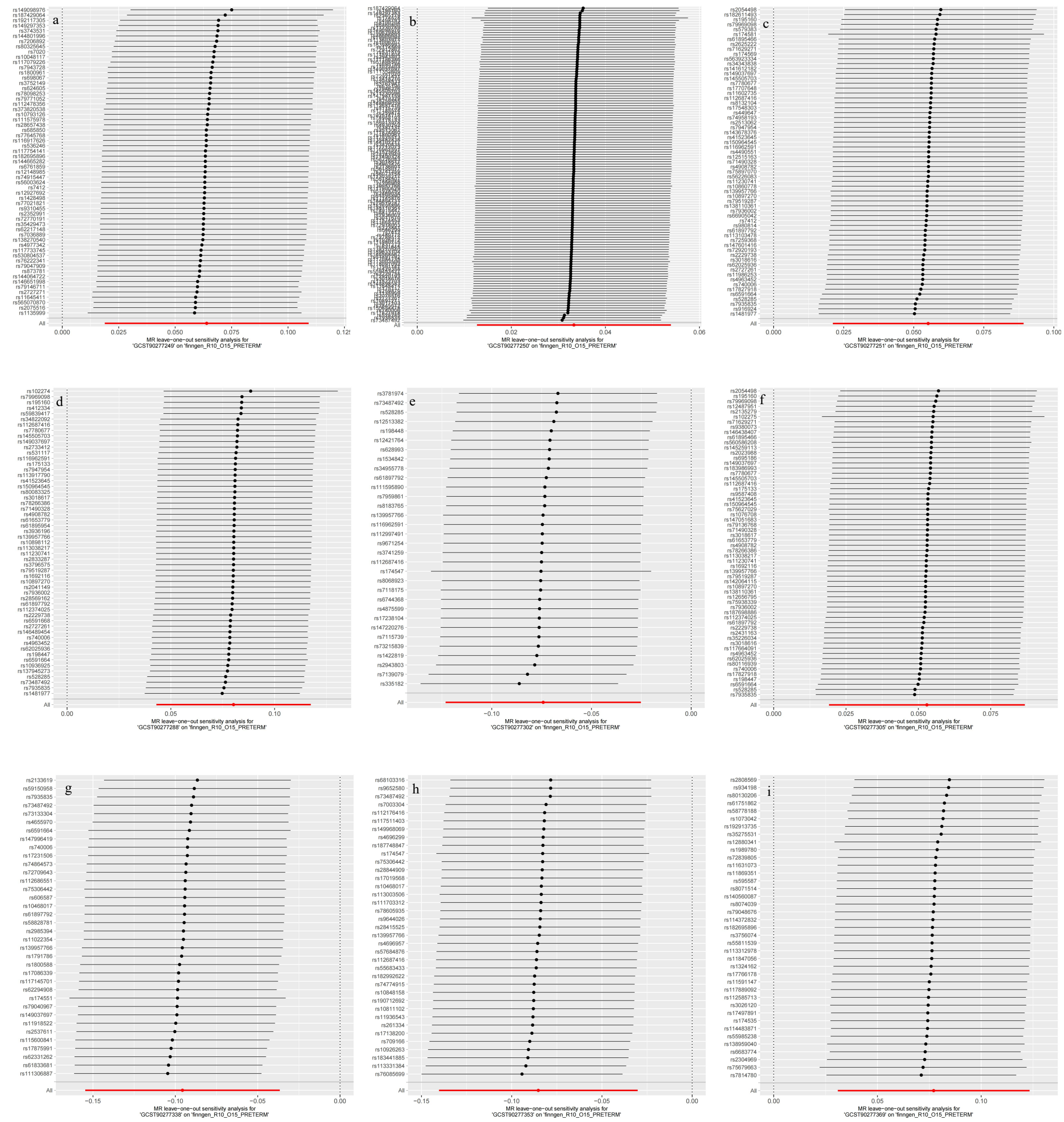
a
MR leave-one-out sensitivity analysis for
'GCST90277249' on 'finngen_R10_O15_PRETERM'
b
MR leave-one-out sensitivity analysis for
'GCST90277250' on 'finngen_R10_O15_PRETERM'
c
MR leave-one-out sensitivity analysis for
'GCST90277251' on 'finngen_R10_O15_PRETERM'
d
MR leave-one-out sensitivity analysis for
'GCST90277288' on 'finngen_R10_O15_PRETERM'
e
MR leave-one-out sensitivity analysis for
'GCST90277302' on 'finngen_R10_O15_PRETERM'
f
MR leave-one-out sensitivity analysis for
'GCST90277305' on 'finngen_R10_O15_PRETERM'
g
MR leave-one-out sensitivity analysis for
'GCST90277338' on 'finngen_R10_O15_PRETERM'
h
MR leave-one-out sensitivity analysis for
'GCST90277353' on 'finngen_R10_O15_PRETERM'
i
MR leave-one-out sensitivity analysis for
'GCST90277369' on 'finngen_R10_O15_PRETERM'

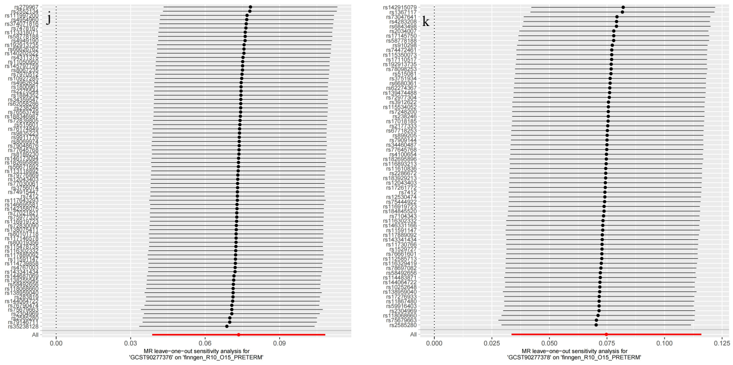

Figure A : Leave-One-Out Cross-Validation Plot for the Association between Circulating Inflammatory Proteins and Preterm Birth: (a) ADA (b) CCL11 (c) CD6 (d) CXCL5 (e) GDNF (f) IL10RB (g) TGFB1 (h) IL18R1 (i) IL24 (j) IL2RB (k) IL4 (l) IL15 (m) LIFR (n) OSM (0) FG21 (p) IL1A (q) CST5 (r) CCL19 . **Figure B** : Leave-One-Out Cross-Validation Plot for the Association between lipidome and Preterm Birth: (**a**) Sterol ester (27:1/20:3) levels (**b**) Sterol ester (27:1/20:4) levels (**c**) Sterol ester (27:1/20:5) levels (**d**) Phosphatidylcholine (16:0_20:5) levels (**e**) Phosphatidylcholine (18:0_20:2) levels (**f**) Phosphatidylcholine (18:0_20:5) levels (**g**) Phosphatidylcholine (O-18:1_18:2) levels (**h**) Phosphatidylethanolamine (O-18:1_18:2) levels (**i**) Sphingomyelin (d34:0) levels (**j**) Sphingomyelin (d40:1) levlipid (**k**) Sphingomyelin (d42:2) levels.
